# Risk Factors for Scrub Typhus, Murine Typhus, and Spotted Fever Seropositivity in Urban Areas, Rural Plains, and Peri-Forest Hill Villages in South India: A Cross-Sectional Study

**DOI:** 10.4269/ajtmh.19-0642

**Published:** 2020-05-26

**Authors:** Carol S. Devamani, Wolf-Peter Schmidt, Koya Ariyoshi, Arumugam Anitha, Saravanan Kalaimani, John A. J. Prakash

**Affiliations:** 1 Department of RUHSA, Christian Medical College, Vellore, India;; 2 Department for Disease Control, London School of Hygiene and Tropical Medicine, London, United Kingdom;; 3 Department of Clinical Medicine, Institute of Tropical Medicine, Nagasaki University, Nagasaki, Japan;; 4 Department of Clinical Microbiology, Christian Medical College, Vellore, India

## Abstract

Scrub typhus and spotted fever group rickettsioses are thought to be common causes of febrile illness in India, whereas they rarely test for murine typhus. This cross-sectional study explored the risk factors associated with scrub typhus, tick-borne spotted fever, and murine typhus seropositivity in three different geographical settings, urban, rural, and hill villages in Tamil Nadu, South India. We enrolled 1,353 participants living in 48 clusters. The study included a questionnaire survey and blood sampling. Blood was tested for *Orientia tsutsugamushi* (scrub typhus), *Rickettsia typhi* (murine typhus), and spotted fever group *Rickettsia* IgG using ELISA. The seroprevalence of scrub typhus, spotted fever, and murine typhus were 20.4%, 10.4%, and 5.4%, respectively. Scrub typhus had the highest prevalence in rural areas (28.1%), and spotted fever was most common in peri-forested areas (14.9%). Murine typhus was more common in rural (8.7%) than urban areas (5.4%) and absent in peri-forested hill areas. Agricultural workers had a higher relative risk for scrub typhus, especially in urban areas. For murine typhus, proximity to a waterbody and owning a dog were found to be major risk factors. The main risk factors for spotted fever were agricultural work and living in proximity to a forest. Urban, rural plains, and hill settings display distinct epidemiological pattern of *Orientia* and rickettsial infections. Although scrub typhus and spotted fever were associated with known risk factors in this study, the findings suggest a different ecology of murine typhus transmission compared with other studies conducted in Asia.

## INTRODUCTION

Scrub typhus is a febrile illness caused by *Orientia tsutsugamushi*, bacteria related but not identical to the genus *Rickettsia* (both are part of the family Rickettsiaceae).^1^ The true rickettsial infections include among others the highly diverse spotted fever group rickettsioses (SFGR) and murine typhus (*Rickettsia typhi*). Scrub typhus is transmitted by the larvae (chiggers) of trombiculid mites which infect mammals as incidental hosts.^2^ Spotted fever is largely transmitted by hard ticks,^3^ whereas murine typhus is transmitted by contact with fleas.^4,5^ Scrub typhus occurs over much of tropical and subtropical East Asia, South Asia, and Southeast Asia.^6^ The disease has recently been identified in Chile^7^ and possibly East Africa.^8^ Spotted fever group rickettsioses and murine typhus occur worldwide.

Scrub typhus and SFGR are thought to be common causes of febrile illness in India.^9–11^ Serological evidence also exists for murine typhus, but is rarely tested for. Delays in disease recognition and treatment may cause a considerable number of preventable deaths across India.^2,9^

This study explored risk factors associated with scrub typhus, spotted fever, and murine typhus seropositivity in three different geographical settings (urban, rural plains, and peri-forested hills) in Tamil Nadu, South India.

## METHODS

### Study design.

**Figure 1.**
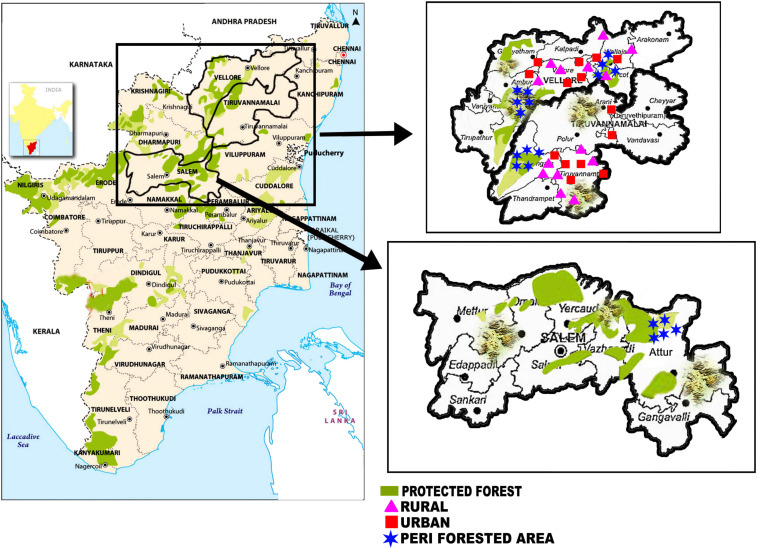
Study area. This figure appears in color at www.ajtmh.org.

The study was a cross-sectional survey, conducted in Vellore, Thiruvannamalai, and Salem districts, all in Tamil Nadu (India). These districts are characterized by three broad geographic areas: urban, rural plains, and rural hill areas up to about 1,000 m elevation. In rural areas (plains and hills), the major crops are rice, sugarcane, sorghum, coconut, pulses, mango, and turmeric. Animal husbandry mainly includes cattle, goats, and poultry. In hill areas, most villages lie at the edge of mountain forests at varying degrees of degradation (peri-forest). In the rural plains, there are isolated patches of secondary forest/dense bush and commercial plantations (e.g., teak and gum tree). The climate is tropical savannah. Monsoon rains occur between June and December. The scrub typhus season approximately lasts from August to February, with a peak from November to January. Scrub typhus occurs sporadically between seasons.^12^ The seasonality of spotted fever and murine typhus has not been studied. The study was conducted in all three geographic settings (urban communities, rural plain villages, and peri-forest hill villages). The study consisted of a questionnaire survey and a blood sample from one individual per enrolled household. Figure 1 shows the areas surveyed.

### Enrollment of communities, households, and participants.

The selection of villages aimed at enrolling high-risk clusters of *Orientia*/rickettsial infection. We did not aim at enrolling a representative sample of the total population of the three districts, as this may have reduced the power of the study to detect risk factors. Selection of study participants was performed in two stages. In the first stage, villages (rural plains and peri-forest hill areas) and urban communities were selected. First, we compiled a list of all communities and villages in the districts with cases of scrub typhus or rickettsial infections admitted to the Christian Medical Collage (CMC), Vellore, in the 5 years before the study. A village or urban community was eligible for enrollment if at least two cases of scrub typhus or rickettsial infection had been admitted during that time. This list comprised 55 urban communities and 53 rural plain villages from two districts (Vellore and Thiruvannamalai) and was dominated by scrub typhus, as the causative agent of more than 90% of all hospitalized *Orientia*/rickettsial infections. For the peri-forest hill villages, this approach had to be modified. Cases occurring in these areas are often treated locally or at government hospitals, presumably because of difficulties in accessing health care at tertiary care centers due to transport problems or financial constraints. Because of these limitations in our knowledge on case numbers at the village level, we chose villages in hill areas where scrub typhus and rickettsial infections were thought to occur based on the experience of local doctors. Within these hill areas of known or presumed endemicity for scrub typhus and rickettsial infections, we purposively selected villages that were easily accessible by road, and added villages from a third district (Salem).

We sampled 30 households per community/village in urban communities and rural plain villages, and 25 households per village in hill areas. Within a chosen community or village, enrollment of households was performed by systematic sampling of every nth household starting from the center of a village with the direction of the walk determined by a flip of a coin (villages situated along a road) or the spin of a bottle (villages/urban communities consisting of clustered houses). The sampling interval was determined by the size of the community/village based on available census data, divided by the required number of participants (30 in urban communities/rural plain villages and 25 in hill villages). The resulting number was rounded down to the next integer. If staff reached the boundary of a village or cluster, they went back to the starting point and chose the opposite direction.

Within a household, one participant was chosen at random from the household members present at the time of the visit. Because of logistical constraints, households without anyone present were not revisited.

### Questionnaire survey and blood sampling.

We administered a questionnaire survey to the selected participants covering sociodemographic, socioeconomic, occupational, geographic, and water/sanitation data. The questionnaire included observation of the household environment and information on livestock and pet animals. The distance to the nearest lake, pond, and forest was estimated using satellite imaging (google maps), with assistance of local residents. A 4-mL venous blood sample, collected in a serum tube, was taken from each participant. The blood was stored in a cool box and transported to the laboratory (CMC, Vellore) either on the same day or within 3 days of collection (Salem district).

### Laboratory analysis.

After collection, blood samples were brought to CMC. Serum was separated from the clot and stored at −70°C until testing. Samples not transported on the same day to CMC were kept in a refrigerator at −4°C after centrifuging the sample to separate serum. IgG antibodies to *O. tsutsugamushi* (56 kDa antigens from Karp, Kato, Gilliam, and TA716 strains) were detected in serum using the scrub typhus IgG ELISA system (InBios International Inc., Seattle, WA). For murine typhus IgG detection, we used the *R. typhi* IgG ELISA (Fuller Laboratory, Fullerton, CA) which covers the species-specific protein rOmp B. For spotted fever IgG, we used the *Rickettsia conorii* ELISA IgG/IgM (Vircell, Granada, Spain).

**Figure 2.**
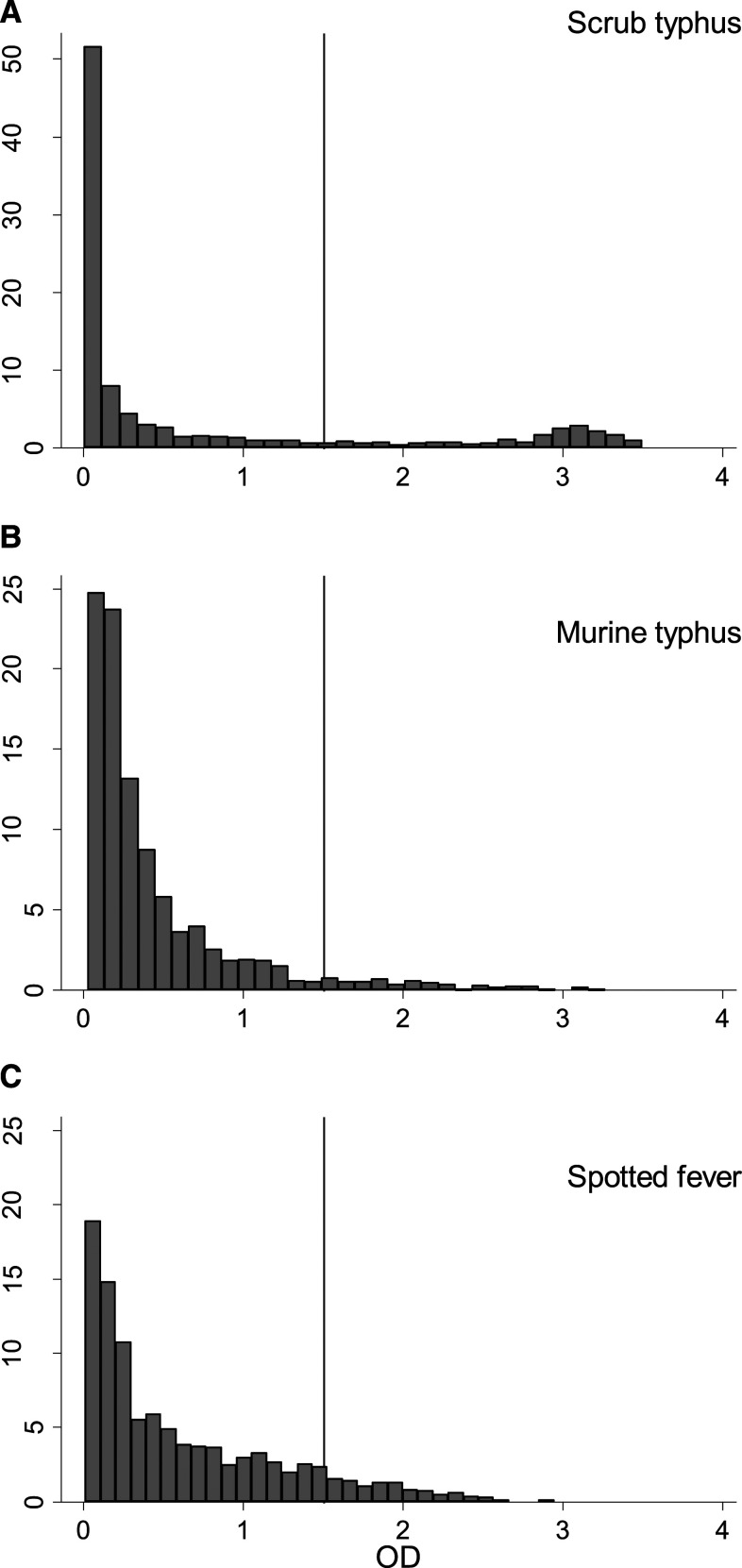
ELISA optical densities.

The commercially available ELISA tests used in this study do not come with cutoff points for the optical density (OD) to define seropositivity that are generally agreed on or recommended by the manufacturers for use in cross-sectional surveys. To define seropositivity, we used an OD cutoff of 1.5 for all three infections. This was based on earlier studies in Vellore district that demonstrated a marked bimodal distribution for scrub typhus IgG in the area.^13,14^ A similar bimodal pattern was found in this study (Figure 2A). The value of 1.5 was chosen as the approximate low point between the two peaks. For murine typhus and spotted fever, there was no clear bimodal pattern in the OD (Figure 2B and C), which made defining a cutoff difficult. In the absence of data on the ELISA OD of IgG antibodies for murine typhus and spotted fever following infection over time, we pragmatically chose the same cutoff as for scrub typhus for both infections (OD = 1.5).

### Sample size.

For each geographic stratum (urban/rural/hill), we aimed at determining IgG seroprevalence with a margin of error of 5%. Assuming a seroprevalence of 20% resulted in a crude sample size of 246 people per stratum. We applied a design effect of 2, resulting in an intended sample size of 492 per stratum.

### Statistical analysis.

All analyses were carried out in STATA 14 (StataCorp LLC, College Station, TX). The primary outcome for the study was seropositivity for scrub typhus, murine typhus, and spotted fever. Prevalence estimates and CIs were calculated using the STATA svy: proportion command with logit CIs. Coinfection with two pathogens was defined as an individual being IgG seropositive for both pathogens.

Prevalence risk ratios were calculated using Poisson regression. CIs were adjusted for the binomial distribution of the data and clustering at the community/village level using robust standard errors. *P*-values for interaction terms were calculated using likelihood ratio tests. Multivariable analysis was carried out including variables that were associated with the outcomes based on effect size and biological plausibility. Intra-class correlation coefficients (ICCs) for clustering of seroprevalence at the community/village level were calculated using the *loneway* command in STATA.

### Ethics.

The study was approved by CMC’s Institutional Review Board, IRB no. 9369. Written consent was obtained from all adult participants. Written or verbal assent was obtained from minors, alongside written consent from their parents/guardians.

## RESULTS

We enrolled 1,353 participants from 16 urban communities, 17 rural plain villages, and 15 peri-forest hill villages (Table 1). Of these, 63% were female and 71% were older than 30 years. Using an ELISA cutoff of 1.5 OD for all pathogens, the overall seroprevalence was highest for scrub typhus, followed by spotted fever and murine typhus (Table 1). Scrub typhus had the highest seroprevalence of all pathogens in urban areas and rural plains. Spotted fever had a higher seroprevalence in hill villages than in urban and rural plain areas. No participant from hill villages was seropositive for murine typhus (Table 1).

**Table 1:** Univariable analysis of risk factors

	*N*	Scrub typhus			Murine typhus			Spotted fever		
		IgG seroprevalence, %	Risk ratio	95% CI	IgG seroprevalence, %	Risk ratio	95% CI	IgG seroprevalence, %	Risk ratio	95% CI
Total	**1,353**	**20.4**		**16.0–25.7**	**5.4**		**3.5–8.2**	**10.4**		**7.4–14.5**
Gender										
Male	505	17.2	Ref		3.6	Ref		10.9		
Female	848	**22.3**	**1.3**	**1.0–1.7**	**6.5**	**1.8**	**1.0–3.3**	10.1	0.9	0.7–1.3
Age-group (years)										
0–20	94	13.8	Ref		3.2	Ref		3.2	Ref	
21–30	294	9.5	0.7	0.4–1.1	3.7	1.2	0.4–3.8	10.2	3.2	1.2–8.8
31–40	320	17.5	1.3	0.7–2.3	6.3	2.0	0.6–6.0	9.4	2.9	1.0–9.0
41–50	263	23.6	1.7	1.0–3.0	6.1	1.9	0.6–5.8	13.3	4.2	1.5–11.9
51–60	229	30.1	2.2	1.2–4.1	6.1	1.9	0.7–5.2	12.2	3.8	1.3–11.7
> 60	153	31.4	2.3	1.2–4.4	5.9	1.8	0.7–4.8	9.8	3.1	0.9–10.4
Geography										
Urban	427	15.5	Ref	–	5.4	Ref		3.8	Ref	
Rural	576	**28.1**	**1.8**	**1.1–2.9**	**8.7**	**1.6**	**0.7–3.5**	12.7	3.4	1.8–6.4
Peri-forested	350	13.7	0.8	0.5–1.7	0.0	–	–	**14.9**	**4.0**	**1.9–8.3**
Educational level										
None	346	21.7	Ref	–	5.2	Ref	–	19.4	Ref	
Primary	268	33.2	1.5	1.0–2.3	7.8	1.5	0.7–3.3	12.7	0.7	0.4–1.1
Middle	291	15.8	0.7	0.4–1.2	3.1	0.6	0.3–1.3	7.2	0.4	0.2–0.6
High school	321	16.5	0.8	0.5–1.1	6.2	1.2	0.6–2.5	5.6	0.3	0.2–0.5
Graduate	126	9.5	0.4	0.2–0.8	4.0	0.8	0.3–2.3	0.8	0.04	0.0–0.3
House type										
Reinforced cement concrete	904	21.0	Ref		5.3	Ref		9.3	Ref	
M sheet	380	16.7	1.1	0.8–1.6	5.5	1.0	0.5–1.7	12.1	0.8	0.5–1.1
Thatched hut	68	22.1	1.2	0.7–2.0	5.9	1.1	0.4–2.9	16.2	1.3	0.7–2.6
Roof material										
Concrete/tiled	1,111	20.1	Ref		5.0	Ref		9.4	Ref	
Thatched	242	21.9	1.1	0.7–1.6	7.0	1.4	0.8–2.5	15.3	1.6	1.0–2.7
House floor material										
Cemented/tiled/mosaic	1,212	21.0	Ref		5.4	Ref		9.0	Ref	
Cow dung/mud	140	15.7	0.7	0.5–1.2	5.0	0.9	0.3–2.5	22.9	2.5	1.7–3.8
Agricultural work										
No	717	17.2	Ref		5.4	Ref		5.3	Ref	
Yes	636	**24.1**	**1.4**	**1.0–1.9**	5.4	1.0	0.5–1.8	**16.2**	**3.1**	**2.0–4.6**
Cultivates rice										
No	752	18.1	Ref		5.3	Ref		5.9	Ref	
Yes	601	23.3	1.3	0.9–1.8	5.5	1.0	0.5–2.0	16.1	2.8	1.7–4.4
Cultivates sugarcane										
No	930	20.9	Ref		7.0	Ref		8.6	Ref	
Yes	423	19.4	0.9	0.6–1.4	1.9	0.3	0.1–0.9	14.4	1.7	0.9–3.0
Cuts grass for fodder										
No	805	19.9	Ref		6.1	Ref		7.1	Ref	
Yes	548	21.2	1.1	0.7–1.5	4.4	0.7	0.4–1.4	15.3	2.2	1.3–3.6
Collects firewood										
No	1,094	20.4	Ref		5.5	Ref		8.7	Ref	
Yes	259	20.5	1.0	0.6–1.7	5.0	0.9	0.4–2.1	17.8	2.0	1.4–3.0
Forest within 300 m										
No	1,014	22.2	Ref		6.4	Ref		6.4	Ref	
Yes	339	15.0	0.7	0.4–1.1	2.4	0.4	0.1–1.4	**22.4**	**3.5**	**2.2–5.6**
Owns vegetable patch										
No	528	19.5	Ref		6.4	Ref		5.5	Ref	
Yes	824	21.0	1.1	0.7–1.6	4.7	0.7	0.3–1.6	13.6	2.5	1.5–4.2
Lake/pond within 300 m										
No	1,046	20.1	Ref		3.2	Ref		11.1	Ref	
Yes	307	21.5	1.1	0.7–1.7	**13.0**	**4.1**	**2.1–8.1**	8.1	0.7	0.4–1.2
House yard has cement floor										
No	131	26.7	Ref		15.3	Ref		10.7	Ref	
Yes	1,221	19.7	0.7	0.4–1.2	4.3	0.3	0.1–0.6	10.4	1.0	0.6–1.6
Walls										
Plastered	1,177	23.5	Ref		5.8	Ref		8.7	Ref	
Non-plastered	176	28.6	0.7	0.4–1.1	2.8	0.5	0.2–1.5	22.2	2.6	1.7–3.9
Cooking place outside										
No	1,055	18.6	Ref		4.9	Ref		9.5	Ref	
Yes	291	26.8	1.4	1.0–2.0	6.9	1.4	0.7–2.7	13.8	1.5	1.0–2.2
Sewage connection										
No	1,005	20.9	Ref		6.5	Ref		12.1	Ref	
Yes	348	19.0	0.9	0.5–1.6	2.3	0.4	0.1–1.2	5.5	0.4	0.2–1.0
Septic tank										
No	665	23.3	Ref		4.8	Ref		13.5	Ref	
Yes	688	17.6	0.8	0.5–1.1	6.0	1.2	0.7–2.2	7.4	0.5	0.3–0.9
Toilet in house/compound										
No	810	20.9	Ref		4.0	Ref		11.7	Ref	
Yes	542	19.7	0.9	0.7–1.4	7.6	1.9	1.1–3.3	8.5	0.7	0.4–1.2
Pet cat										
No	1,306	20.2	Ref		5.4	Ref		10.7	Ref	
Yes	47	25.5	1.3	0.7–2.2	6.4	1.2	0.4–3.3	2.1	0.2	0.0–1.4
Pet dog										
No	896	20.4	Ref		2.8	Ref		9.5	Ref	
Yes	456	20.2	1.0	0.7–1.5	**10.5**	**3.8**	**2.2–6.4**	12.3	1.3	0.9–1.9
Owns cows										
No	835	18.0	Ref		4.6	Ref		7.9	Ref	
Yes	516	**24.2**	**1.3**	**1.0–1.8**	6.8	1.5	0.9–2.6	**14.5**	**1.8**	**1.3–2.6**
Owns goats										
No	1,134	20.7	Ref		5.6	Ref		8.4	Ref	
Yes	217	18.9	0.9	0.6–1.3	4.6	0.8	0.4–1.8	**21.2**	**2.5**	**1.7–3.7**
Owns poultry										
No	1,129	18.6	Ref		4.6	Ref		9.0	Ref	
Yes	223	**29.6**	**1.6**	**1.1–2.4**	**9.4**	**2.0**	**1.2–3.6**	**18.0**	**2.0**	**1.2–3.4**
History of fever in the last 6 months										
No	1,299	19.9	Ref		5.3	Ref		10.5	Ref	
Yes	52	32.7	1.6	1.0–2.8	7.7	1.4	0.6–3.3	9.6	0.9	0.4–2.0

Values in bold indicate *P* values < 0.05.

There was some evidence that pairwise dual seropositivity among any of the three pathogens was more common than expected by chance. Dual seropositivity of scrub typhus and spotted fever affected 3% of individuals (*n* = 40, versus 28.8 expected by chance). Dual seropositivity of scrub typhus and murine typhus was observed in 1.9% of participants (*n* = 26, versus 14.9 expected). Murine typhus/spotted fever dual seropositivity was very rare, affecting only 0.9% of participants (*n* = 12, versus 7.6 expected).

In univariable analysis, females were more commonly seropositive for scrub typhus and murine typhus than males. For spotted fever, there was no difference. Risk factors markedly associated with scrub typhus seropositivity in univariable analysis included older age, lower education level, agricultural work, an outside cooking place, cow ownership, poultry ownership, and a history of fever in the last 6 months.

**Figure 3.**
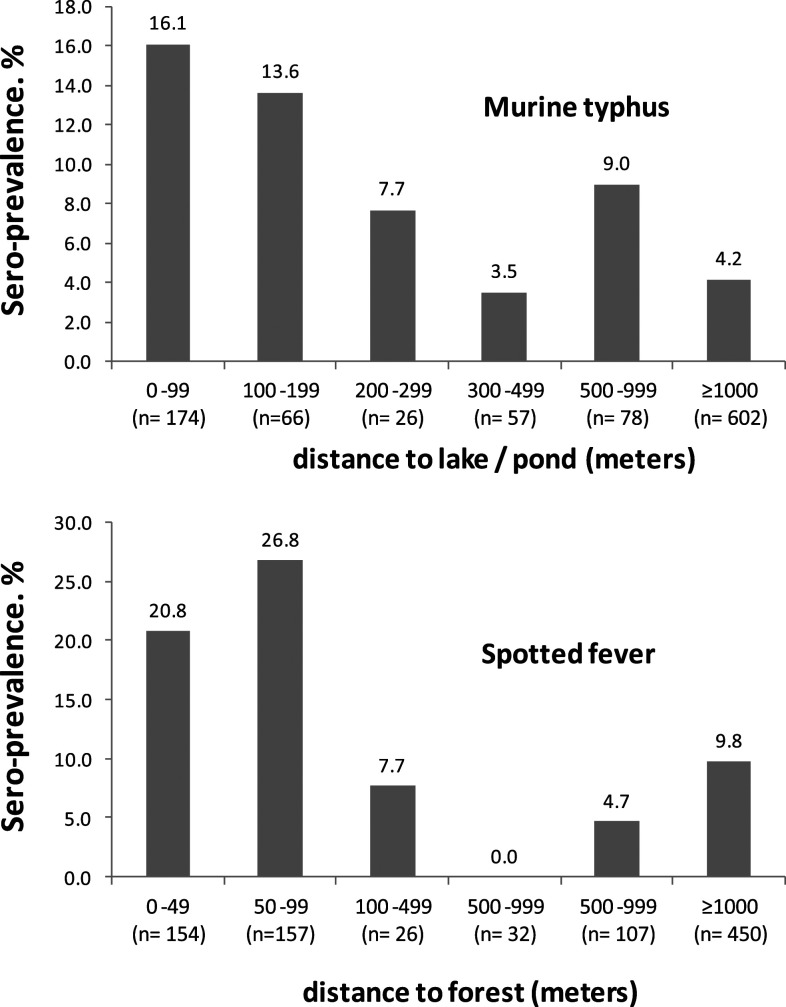
(**A**) Association between distance to the nearest lake or pond and murine typhus seroprevalence (excluding hill villages). (**B**) Association between distance to forest and spotted fever seroprevalence (excluding urban areas).

Risk factors markedly associated with murine typhus seropositivity in univariable analysis included absence of a forest within 1 km, an uncemented house yard, and dog and poultry ownership. Seroprevalence appeared to be particularly high for participants living within 300 m of a pond or lake (Figure 3A). For this graph, peri-forest hill areas were excluded as the prevalence of murine typhus was zero.

In univariable analysis, there was a marked association between spotted fever seropositivity and lower education level. Other factors associated with spotted fever were thatched house roof, mud house floor, agricultural work, grass cutting for fodder, firewood collection, proximity to forest, ownership of a vegetable patch, unplastered walls, outside cooking, and ownerships of cows, goats, and poultry. Figure 3B shows the association between distance to forest and spotted fever seroprevalence, confirming proximity to a forest as a strong risk factor.

In further stratified analysis, we explored whether there was effect modification of these risk factors by geography (urban/rural/hill). The association between farming practices (agriculture, and cow and poultry ownership) and scrub typhus was much stronger in urban areas than in rural and hill villages where seroprevalence was overall higher (Table 2). For agricultural work, the test for interaction (urban versus rural + hill) showed a *P*-value of < 0.001. For murine typhus, the risk ratios for pet dog ownership and proximity to lake/pond were somewhat stronger in urban than in rural areas (Table 3, test for interaction *P* = 0.36 and *P* = 0.39). For spotted fever (Table 4), the association with cutting grass was somewhat stronger in hill areas (test for interaction *P* = 0.34).

**Table 2:** Scrub typhus effect modification by geography

	Urban				Rural				Peri-forest			
	*N*	IgG seroprevalence, %	Risk ratio	95% CI	*N*	IgG seroprevalence, %	Risk ratio	95% CI	*N*	IgG seroprevalence, %	Risk ratio	95% CI
Gender												
Male	157	10.2	Ref	–	204	26.0	Ref	–	144	12.5	Ref	–
Female	270	18.5	1.8	1.0–3.3	372	29.3	1.1	0.8–1.6	206	14.6	1.2	0.7–2.0
Age-group (years)												
0–20	36	8.3	Ref	–	41	14.6	Ref	–	17	23.5	Ref	–
21–30	90	4.4	0.5	0.1–2.4	109	14.7	1.0	0.5–1.9	95	8.4	0.4	0.2–0.8
31–40	114	9.7	1.2	0.3–5.0	125	28.8	2.0	0.8–4.7	81	11.1	0.5	0.2–1.0
41–50	83	21.7	2.6	0.7–9.4	118	33.9	2.3	1.1–5.0	62	6.5	0.3	0.1–0.9
51–60	61	24.6	3.0	0.613.9	114	32.5	2.2	0.9–5.5	54	31.5	1.3	0.5–3.5
> 60	43	34.9	4.2	1.1–15.7	69	39.1	2.7	1.1–6.7	41	14.6	0.6	0.2–2.1
Education level												
None	32	28.1	Ref	–	129	31.0	Ref	–	185	14.1	Ref	–
Primary	85	28.2	1.0	0.7–1.4	158	39.9	1.3	0.8–2.1	25	8.0	0.6	0.1–2.3
Middle	104	11.5	0.4	0.2–0.7	97	19.6	0.6	0.3–1.2	90	16.7	1.2	0.5–3.1
High school	154	11.7	0.4	0.3–0.7	132	23.5	0.8	0.4–1.3	35	11.4	0.8	0.3–2.4
Graduate	52	5.8	0.2	0.1–0.6	59	13.6	0.4	0.2–0.9	15	6.7	0.5	0.1–2.3
Agricultural work												
No	394	12.9	Ref	–	274	24.5	Ref	–	49	10.2	Ref	
Yes	33	45.5	3.5	2.4–5.2	302	31.5	1.3	0.9–1.8	301	14.3	1.4	0.4–4.5
Owns cows												
No	395	14.4	Ref	–	245	26.9	Ref		195	13.9	Ref	
Yes	32	28.1	1.9	1.0–3.8	329	28.9	1.1	0.8–1.5	155	13.6	1.0	0.5–2.1
Owns poultry												
No	418	15.3	Ref		370	26.8	Ref	–	341	13.8	Ref	
Yes	9	22.2	1.5	0.3–6.8	205	30.7	1.1	0.7–1.8	9	11.1	0.8	0.2–2.7

**Table 3:** Murine typhus effect modification by geography

	Urban				Rural			
	*N*	IgG seroprevalence, %	Risk ratio	95% CI	*N*	IgG seroprevalence, %	Risk ratio	95% CI
Gender								
Male	270	5.9	Ref	–	372	10.5	Ref	–
Female	157	4.5	1.3	0.4–4.9	204	5.4	1.9	1.0–3.7
Age-group (years)								
0–20	36	2.8	Ref	–	41	4.9	Ref	–
21–30	90	5.6	2.0	0.3–11.5	109	5.5	1.1	0.2–5.7
31–40	114	4.4	1.6	0.2–16.3	125	12.0	2.5	0.7–8.1
41–50	83	9.6	3.5	0.6–20.6	118	6.8	1.4	0.3–6.2
51–60	61	1.6	0.6	0.0–10.0	114	11.4	2.3	0.8–6.6
> 60	43	7.0	2.5	0.5–12.8	69	8.7	1.8	0.6–5.5
Education level								
None	32	3.1	Ref	–	129	13.2	Ref	–
Primary	85	8.2	2.6	0.2–28.7	158	8.9	0.7	0.3–1.5
Middle	104	4.8	1.5	0.2–11.5	97	4.1	0.3	0.1–0.8
High school	154	5.2	1.7	0.2–16.4	132	9.1	0.7	0.3–1.5
Graduate	52	3.9	1.2	0.1–15.8	59	5.1	0.4	0.1–1.6
Lake/pond within 300 m								
No	313	2.9	Ref	–	414	5.8	Ref	–
Yes	114	12.3	4.3	1.4–13.3	162	16.1	2.8	1.3–5.8
House yard has cement floor								
No	31	22.6	Ref	–	25	–	Ref	–
Yes	396	4.0	0.2	0.0–0.9	324	–	0.4	0.2–0.8
Pet dog								
No	270	2.6	Ref	–	331	5.4	Ref	–
Yes	157	10.2	3.9	1.4–10.8	244	13.1	2.4	1.3–4.6
Owns poultry								
No	418	5.5	Ref	–	370	7.8	Ref	–
Yes	9	0.0	–	–	205	10.2	1.3	0.7–2.4

**Table 4:** Spotted fever effect modification by geography

	Urban				Rural				Peri-forest			
	*N*	IgG seroprevalence, %	Risk ratio	95% CI	*N*	IgG seroprevalence, %	Risk ratio	95% CI	*N*	IgG seroprevalence, %	Risk ratio	95% CI
Gender												
Male	157	2.6	1	–	204	12.8	1	–	144	17.4	1	–
Female	270	4.4	1.7	0.5–5.6	372	12.6	1.0	0.7–1.5	206	13.1	0.8	0.4–1.3
Age-group (years)												
0–20	36	2.8	Ref		41	2.4	Ref	–	17	5.9	Ref	–
21–30	90	3.3	1.2	0.3–5.3	109	11.9	4.9	0.6–38.0	95	14.7	2.5	0.6–11.4
31–40	114	5.3	1.9	0.2–17.0	125	11.2	4.6	0.7–29.3	81	12.4	2.1	0.3–14.3
41–50	83	1.2	0.4	0.0–6.4	118	17.8	7.3	1.1–49.5	62	21.0	3.6	0.7–19.5
51–60	61	6.6	2.4	0.2–22.4	114	14.0	5.8	0.8–40.3	54	14.8	2.5	0.4–15.3
> 60	427	3.8	0.8	0.1–12.1	69	11.6	4.8	0.6–41.0	41	14.6	2.5	0.4–15.7
Education level												
None	32	3.1	Ref		129	20.2	Ref		185	21.6	Ref	–
Primary	85	8.2	2.6	0.8–8.2	158	16.5	0.8	0.4–1.6	25	4.0	0.2	0.0–1.4
Middle	104	2.9	0.9	0.1–6.4	97	11.3	0.6	0.3–1.2	90	7.8	0.4	0.2–0.7
High school	154	3.3	1.0	0.2–6.6	132	6.8	0.3	0.2–0.6	35	11.4	0.5	0.2–1.5
Graduate	52	0.0	–	–	59	1.7	0.1	0.0–0.5	15	0.0	–	–
Agricultural work												
No	394	3.6	1	–	274	8.0	1	–	49	4.1	1	–
Yes	33	6.1	1.7	0.3–10.3	302	17.0	2.1	1.4–3.3	301	16.6	4.1	1.6–10.5
Forest within 300 m												
No	425	3.8	Ref	–	428	9.6	Ref	–	161	5.0	Ref	–
Yes	2	0.0	–	–	148	21.6	2.3	1.2–4.3	189	23.3	4.7	2.3–9.4
Owns vegetable Patch												
No	393	4.1	Ref	–	133	9.8	Ref	–	2	0.0	Ref	–
Yes	34	0.0	–	–	442	13.6	1.4	0.7–2.8	348	14.9	–	–
Collects firewood												
No	424	3.3	Ref	–	405	11.6	Ref	–	265	12.8	Ref	–
Yes	3	66.7	20.2	11.9–34.3	171	15.2	1.3	0.8–2.1	85	21.2	1.7	0.9–2.9
Cuts grass for fodder												
No	419	3.3	Ref		327	12.2	Ref	–	59	5.1	Ref	–
Yes	8	25.0	7.5	3.1–18.0	249	13.2	1.1	0.6–1.9	291	16.8	3.3	1.4–7.8
Owns cows												
No	395	3.0	Ref	–	245	11.8	Ref		195	12.8	Ref	–
Yes	32	12.5	4.1	1.1–15.1	329	13.4	1.1	0.8–1.6	155	17.4	1.4	0.8–2.2
Owns goats												
No	414	3.6	Ref	–	456	11.6	Ref	–	264	10.2	Ref	–
Yes	13	7.7	2.1	0.4–12.0	119	16.8	1.4	0.9–2.4	85	29.4	2.9	1.7–4.7
Owns poultry												
No	418	3.6	Ref	–	370	9.5	Ref	–	341	15.0	Ref	–
Yes	9	11.1	3.1	0.3–28.9	205	18.5	2.0	1.1–3.5	9	11.1	0.7	0.2–2.5

In multivariable analysis (Table 5), the following variables were included: gender, age, geography, education, agricultural work, owning a cow, owning poultry, owning goat, owning pet dog, collecting grass, collecting firewood, own vegetable patch, forest within 300 m, lake/pond within 300 m, and house yard with cement floor. Older age and agricultural work remained markedly associated with scrub typhus. There was some evidence that owning a pet dog and proximity to lake or pond remained associated with murine typhus (approximately doubling the prevalence risk). Level of education remained a strong risk factor for spotted fever, alongside agricultural work and to a lesser degree owning a goat. Cutting grass was no longer associated with spotted fever after adjustment in multivariable analysis. The ICC for clustering at the village/community level was 0.14 for scrub typhus, 0.08 for murine typhus (excluding hill villages where the prevalence was zero), and 0.11 for spotted fever. Values mentioned in bold in Tables 1 and 5 are those found significant by univariable and multivariable analysis.

**Table 5:** Multivariable analysis

	Scrub typhus		Murine typhus		Spotted fever	
	Risk ratio	95% CI	Risk ratio	95% CI	Risk ratio	95% CI
Gender						
Male	Ref	–	Ref	–	Ref	–
Female	1.1	0.9–1.4	1.8	0.9–2.9	0.9	0.6–1.1
Age (per 10-year increase)	1.21	1.10–1.32	1.07	0.89–1.29	0.98	0.87–1.11
Geography						
Urban	Ref	–	Ref	–	Ref	–
Rural	1.3	0.8–2.0	1.4	0.8–2.7	1.4	0.6–3.2
Peri-forested	0.7	0.4–1.3	0.0	0.0	1.3	0.5–3.3
Educational level						
None	Ref	–	Ref	–	Ref	–
Primary	1.5	1.1–2.1	1.4	0.7–3.0	1.1	0.7–1.7
Middle	1.0	0.6–1.6	0.8	0.4–1.7	0.6	0.4–0.9
High school	1.1	0.7–1.6	1.1	0.5–2.2	0.5	0.3–1.0
Graduate	0.8	0.4–1.4	0.8	0.2–2.7	0.1	0.0–0.5
Agricultural work						
No	Ref	–	–	–	Ref	–
Yes	**1.5**	**1.1–2.1**	–	–	**1.9**	**1.2–2.9**
Owns cows						
No	Ref	–	–	–	Ref	–
Yes	1.1	0.8–1.6	–	–	1.0	0.7–1.3
Owns poultry						
No	Ref	–	Ref	–	Ref	–
Yes	1.1	0.8–1.6	1.1	0.7–1.7	1.3	0.7–2.4
Owns goats						
No	–	–	–	–	Ref	
Yes	–	–	–	–	1.3	0.9–1.9
Pet dog						
No	–	–	Ref	–	–	–
Yes	–	–	**2.0**	**0.8–4.8**	–	–
Cuts grass for fodder						
No	–	–	–	–	Ref	–
Yes	–	–	–	–	0.8	0.5–1.3
Collects firewood						
No	–	–	–	–	Ref	–
Yes					1.0	0.7–1.3
Owns vegetable patch						
No	–	–	–	–	Ref	–
Yes	–	–	–	–	1.0	0.5–2.0
Forest within 300 m						
No	–	–	–	–	Ref	–
Yes	–	–	–	–	**2.1**	**1.3–3.3**
Lake/pond within 300 m						
No	–	–	Ref	–	–	–
Yes		–	**2.2**	**0.9–5.3**	–	–
House yard has cement floor	–					
No	–	–	Ref	–	–	–
Yes	–	–	0.5	0.2–1.1	–	–

Values in bold indicate *P* values < 0.05.

## DISCUSSION

This cross-sectional study explored the seroprevalence of scrub typhus, spotted fever, and murine typhus, and associated risk factors. Scrub typhus had the highest prevalence in rural areas, whereas spotted fever was most common in peri-forested areas. Murine typhus was more common in rural than in urban areas and absent in peri-forested areas.

Agricultural workers had a higher risk for scrub typhus, especially in urban areas where the overall risk was lower. Older age was also found to be a risk factor for scrub typhus, but not for murine typhus and spotted fever. For murine typhus, proximity to a lake/pond was found to be a major risk factor along with owning a dog. The main risk factors for spotted fever were agricultural work and proximity to a forest.

In line with these findings, studies conducted in Laos, Korea, Indonesia, China, and Malaysia identified female gender, older age, and farming work as risk factors, with the infection being generally more common in rural areas.^5,15–22^ Urban areas have, however, long been known to harbor scrub typhus in endemic countries.^23^ Rickettsioses other than scrub typhus are also not uncommon in urban areas, as reported by Tshokey and others^24^.

A prospective case–control study by George and others^11^ conducted in 2013 showed that farming activities were a risk factor for acquiring scrub typhus in adults. In further studies, the risk of scrub typhus was associated with being female, age > 60 years,^13^ in agricultural laborers, being bare-chested at home, and living in dwellings adjacent to scrub land.^12^ A pediatric case–control study suggested scrub typhus is more likely in children who have pets and stay in houses less than 100 m from a waterbody and bushes within 5 m.^25^ The strong association of agricultural work with scrub typhus in urban areas in our study suggests that infestation with mites may often take place away from urban areas, for example, in those who own agricultural land and go to rural areas for cultivation.

In urban Laos, proximity to market places and dense urban communities were risk factors.^15^ However, in our setting, murine typhus was found to be more common in rural areas with possible risk factors including proximity to waterbodies and ownership of a dog, both of which are common in rural settings. Although fleas transmitting murine typhus have classically been shown to be carried by cats, rats, and opossums,^26^ studies have also shown dogs to carry cat fleas (*Ctenocephalides felis*) and to be infected with *R. typhi*.^4,27^ The association with proximity to waterbodies may, however, also suggest a role of rodents in maintaining transmission. A similar association with waterbodies has been found for leptospirosis, for which rodents are implicated in transmission.^28^ Therefore, our findings suggest that the possible ecology of murine typhus is different in our setting as compared with others areas in Asia such as Laos.^15^

Little is known about the occurrence of tick-borne SFGR in India.^3^ Only a few case series have been published.^10,29–32^ In our study, spotted fever was found to be common among those living in proximity to a forest and agricultural workers, which is in line with studies conducted in Malaysia, Thailand, Indonesia, and Brazil.^33–41^

The study is mainly limited by the pragmatic choices for ELISA cutoff points, the possibility of cross-reactivity among the three infections, and the approach to enroll participants. Seroprevalence estimates depend on the chosen cutoff point for the OD. Furthermore, the interpretation of seropositivity in an individual depends on the average duration an individual remains seropositive after infection. For example, if infected individuals remain seropositive for life, then seropositivity reflects the cumulative lifetime risk of asymptomatic (serological) or symptomatic infection. However, IgG positivity may only persist for a limited amount of time. A study in Vellore district suggested that most people may revert to seronegativity within 3 years after scrub typhus infection.^42^ However, the bimodal distribution of OD values and the near-linear increase in seropositivity with age may suggest that scrub typhus IgG may persist for many years after infection, at least in a sizable proportion of infected.^14^ Similar data are not available for spotted fever and murine typhus. This lack of data on IgG response following infection makes the choice for a suitable OD cutoff and the interpretation of seroprevalence estimates difficult, in particular for murine typhus and spotted fever. In this study, seroprevalence may mean little more than the proportion of the population that has been infected within months or years before the survey. These uncertainties in defining a valid cutoff and in the duration of IgG antibody persistence need to be borne in mind when interpreting absolute prevalence estimates. They should, however, play a lesser role when calculating relative effect measures such as the prevalence risk ratio for different risk factors.

Cross-reactivity among *Orientia* and rickettsial antibodies has been mainly studied in ELISA tests for acute infection (IgM).^43^ Data on cross-reactivity of IgG antibodies are limited. Murine typhus and spotted fever group rickettsiosis antibodies may be particularly prone to cross-react with scrub typhus antigens as these are caused by related organisms. A study from an urban setting in Laos jointly examined the prevalence of positive scrub typhus and murine typhus IgG antibodies (based on ELISA tests) in the general population, demonstrating different geographic risk factors for the two infections.^15^ In this study of 2002 people, 314 were found to be positive only for scrub typhus antibodies, 360 positive only for murine typhus antibodies, whereas 80 were positive for both antibodies. The expected number of dual positives assuming independence between the two infections would have been 86 cases—very similar to the observed 80. If cross-reactivity were substantial, then one would expect a higher proportion of participants to be positive for both infections. This finding suggests that cross-reactivity, while of clinical relevance, especially in acute cases, may be of lesser importance in serological surveys using IgG. In the present study, dual seropositivity was slightly more common than expected, especially between scrub typhus and rickettsial infections under study. However, this may also represent true dual seropositivity due to overlapping risk factors such as agricultural work (scrub typhus and spotted fever) and rural plain geography (scrub typhus and murine typhus). If cross-reactivity were a substantial problem, then one would have expected a far higher proportion of dual seropositivity among the three pathogens. Furthermore, if there was relevant cross-reactivity between spotted fever and murine typhus antibodies, we should have found some cases of murine typhus seropositivity to occur in hill areas where spotted fever was common.

Finally, the study lacked a formal sampling frame for enrolling villages and individuals within clusters. The aim of the study was to study risk factors for the three pathogens which are easier and more precise to do if the study population is at high risk. We therefore chose to enroll presumed high-risk clusters of *Orientia*/rickettsial infection. Because of the predominance of scrub typhus in the area, the sample was probably biased toward enrolling scrub typhus clusters, and not necessarily represents high-risk murine typhus and spotted fever clusters, which often may not be diagnosed using reliable tests. The enrollment of peri-forest hill villages was largely performed purposively, because of logistical constraints. Enrollment of participants within households was restricted to those present at the time of the survey, which is likely to bias the sample toward those being at home more often, such as women and the elderly. People commuting to work and those working away in fields may have been underrepresented. Because of the nonrandom enrollment of clusters and individuals, absolute seroprevalence estimates in this study may not be generally applicable to similar settings in South India. There is, however, little reason to assume that the study procedures introduced substantial bias to the risk ratio estimates.

Testing a large number of risk factors as performed here may lead to associations arising by chance. Nevertheless, it seems justified to conclude that in South India, urban, rural plains, and hill settings display distinct epidemiological pattern of *Orientia* and rickettsial infections. Although scrub typhus and spotted fever were associated with known risk factors in this study, the study suggests a different ecology of murine typhus transmission compared with other studies done in Asia, possibly involving domestic animals. Future studies should 1) explore vector–host relationships especially for murine typhus, 2) provide a more complete clinical picture for spotted fever and murine typhus which are rarely diagnosed in India, and 3) determine for all three infections studied here the longevity of IgG antibodies following infection, which will allow a more meaningful interpretation of seroprevalence data.
